# Role Played by Paraoxonase-2 Enzyme in Cell Viability, Proliferation and Sensitivity to Chemotherapy of Oral Squamous Cell Carcinoma Cell Lines

**DOI:** 10.3390/ijms24010338

**Published:** 2022-12-25

**Authors:** Roberto Campagna, Alessia Belloni, Valentina Pozzi, Alessia Salvucci, Valentina Notarstefano, Lucrezia Togni, Marco Mascitti, Davide Sartini, Elisabetta Giorgini, Eleonora Salvolini, Andrea Santarelli, Lorenzo Lo Muzio, Monica Emanuelli

**Affiliations:** 1 Department of Clinical Sciences, Polytechnic University of Marche, 60020 Ancona, Italy; 2 Department of Life and Environmental Sciences, Polytechnic University of Marche, 60131 Ancona, Italy; 3 Dentistry Clinic, National Institute of Health and Science of Aging, IRCCS INRCA, 60124 Ancona, Italy; 4 Department of Clinical and Experimental Medicine, University of Foggia, 71122 Foggia, Italy; 5 New York-Marche Structural Biology Center (NY-MaSBiC), Polytechnic University of Marche, 60131 Ancona, Italy

**Keywords:** OSCC, PON2, cell growth, chemosensitivity

## Abstract

Oral squamous cell carcinoma represents the most aggressive and frequent form of head and neck cancer. Due to drug resistance, the 5-year survival rate of patients with advanced disease is less than 50%. In order to identify molecular targets for effective oral cancer treatment, we focused on paraoxonase-2 enzyme. Indeed, based on data previously obtained from preliminary immunohistochemistry and Western blot analyses performed on tissue specimens, the enzyme was found to be upregulated in tumor compared with normal oral mucosa. Therefore, paraoxonase-2 gene silencing was achieved in HSC-3 and HOC621 oral cancer cell lines, and the effect on cell proliferation, viability, apoptosis induction and sensitivity to cisplatin and 5-fluorouracil treatment was evaluated. Fourier Transform InfraRed Microspectroscopy analyzed alterations of cellular macromolecules upon treatment. Enzyme level and cell proliferation were also determined in cisplatin-resistant clones obtained from HOC621 cell line, as well as in parental cells. Reported data showed that paraoxonase-2 knockdown led to a reduction of cell proliferation and viability, as well as to an enhancement of sensitivity to cisplatin, together with the activation of apoptosis pathway. Spectroscopical data demonstrated that, under treatment with cisplatin, oxidative damage exerted on lipids and proteins was markedly more evident in cells down-regulating paraoxonase-2 compared to controls. Interestingly, enzyme expression, as well as cell proliferation were significantly higher in cisplatin-resistant compared with control HOC621 cells. Taken together these results seem to candidate the enzyme as a promising target for molecular treatment of this neoplasm.

## 1. Introduction

Oral cancer develops from any site in the oral cavity, including tongue, lip, gingiva, palate, floor of the mouth or buccal mucosa, with an incidence of more than 300,000 new cases per year. More than 90% of all oral cancers are classified as oral squamous cell carcinoma (OSCC), representing the most aggressive and frequent neoplastic form among oral malignancies in developing countries [1,2,3].

OSCC originates from oral keratinocytes, and main factors able to potentially increase the risk of its incidence are tobacco smoking, alcohol consumption and HPV infection [4]. Prolonged inflammation status, induced by the exposure of oral cavity cell to mutagenic agents, leads to the development of potentially malignant disorders, such as leukoplakia and erythroplakia, that can degenerate into OSCC [5]. Time of diagnosis related to the history of the disease greatly influences both treatment choices and patient prognosis. Indeed, subjects diagnosed with early stage OSCC undergo surgery, radiotherapy or a combination of both treatments, and display a good prognosis, with cure rates ranging between 65 and 80% [4,5]. Unfortunately, due to delay in detection, only one-third of newly diagnosed patients is affected with stage I-II OSCC, with the rest having locally advanced or metastatic disease. Late stage OSCC is treated with surgical removal of primary tumor followed by adjuvant chemotherapy and radiotherapy, while neoadjuvant chemotherapy is recommended for patients affected by unresectable tumors [6,7]. Despite important improvements in the fields of surgery and pharmacological therapy, the 5-year survival rate of patients suffering from advanced OSCC is less than 50%, with frequent recurrence, drug resistance and metastatic spread being the main causes of cancer-related deaths [4].

Current chemotherapeutic drugs, that are used for treatment of advanced or metastatic OSCC, include platinum-based compounds, such as cisplatin (CDDP) and carboplatin, taxanes, as paclitaxel and docetaxel, anthracyclines like adriamycin, epirubicin, pirarubicin, doxorubicin and antimetabolites, namely methotrexate and 5-fluorouracil (5-FU). In clinical practice, patients receive CDDP alone or combined with 5-FU and paclitaxel [8,9]. Upon initial positive response to chemotherapy, tumor acquires drug-resistance, leading OSCC patients to experience early relapse and the onset of metastatic disease. The acquisition of chemo-resistant phenotype could be attributable to cancer stem cell subpopulations, as well as to drug-induced selection of cellular clones refractory to chemotherapeutic treatments [10]. In the light of these considerations, it is crucial to identify molecules that, participating to oral carcinogenesis and influencing tumor cell phenotype, could represent valuable targets to setup effective anticancer therapies.

In this study, we focused our attention on the enzyme paraoxonase-2 (PON2), whose involvement was recently reported in relation to many neoplasms. Together with paraoxonase-1 (PON1) and paraoxonase-3 (PON3), PON2 belongs to the human paraoxonase (PON) family, whose gene members are located within a cluster on chromosome 7 [11]. While PON1 and PON3 are mainly expressed in hepatocytes and subsequently secreted into the serum, where they are associated to circulating high-density lipoproteins, PON2 is expressed in many tissues and displays an intracellular localization [11]. Constitutive enzyme expression was described in both primary and immortalized endothelial cell lines [12], as well as in small intestine [13] and central nervous system [14], where it seems to display an important role among cellular strategies against oxidative stress. Within cell, PON2 is associated with membranes of endoplasmic reticulum [15] and mitochondria [16,17], as well as with nuclear envelope [15] and plasma membrane [18].

PON2 capacity to act as negative regulator towards prooxidative stimuli mainly relies on its ability to reduce levels of reactive oxygen species (ROS) [17]. This effect is particularly evident in mitochondria, where PON2 is strictly associated to coenzyme Q10 within the inner membrane and counteracts the production of superoxide anion during the electron transport chain [16,17]. A recent scientific interest has focused on studying the potential role played by the enzyme in cancer. Reported data clearly demonstrate that PON2 is overexpressed in different tumors, including, bladder cancer (BC) [19], ovarian cancer (OC) [20], pancreatic ductal adenocarcinoma (PDAC) [21], gastric cancer (GC) [22] and skin neoplasms, such as basal cell carcinoma (BCC) and melanoma [23]. In OSCC, the enzyme displayed to be variably expressed both in vivo and in vitro. Moreover, analyses performed on cell lines revealed a potential PON2 contribution to cellular events leading to radioresistance [24]. However, to date, no data were available concerning PON2 dysregulation in OSCC (tumor versus normal oral mucosa), as well as its potential involvement in other aspects related to cancer cell phenotype, such as response to chemotherapy. Preliminary and ongoing analyses, performed on a large cohort of matched cancer and normal tissue specimens and aimed to evaluate the prognostic potential of PON2 in OSCC, revealed a significant enzyme dysregulation. In particular, Western blot and immunohistochemistry demonstrated that PON2 expression levels were markedly higher in OSCC compared to healthy oral mucosa (data not published yet).

In the light of the above evidences, in order to speculate PON2 capacity to participate to mechanisms related to OSCC cell chemosensitivity, short hairpin RNA (shRNA)-mediated knockdown of the enzyme was achieved in both HSC-3 and HOC621 OSCC cell lines, and the effects of PON2 silencing were evaluated on cell proliferation, viability and apoptosis induction, before and after treatment with CDDP and 5-FU, used alone or in combination. Furthermore, Fourier Transform InfraRed Microspectroscopy (FTIRM) was also used to assess the effects of PON2-induced downregulation on sensitivity of OSCC cells to chemotherapeutic treatment. Spectral information was statistically analyzed and correlated with results of cell-based assays in order to identify significant spectroscopic signatures attributable to alterations in the composition of cellular macromolecules. PON2 protein expression and cell viability were then evaluated in chemo-resistant cellular clones selected from HOC621 cell line, as well as in parental cells, by culturing them with CDDP at increasing concentrations.

## 2. Results

### 2.1. Efficiency of PON2 Silencing in HSC-3 and HOC621 Cells

In order to modulate enzyme expression for functional analysis, OSCC cell lines were transfected as reported under Materials and Methods. Upon selection of cellular clones resistant to puromycin, the efficiency of PON2 knockdown was determined at mRNA and protein level, by Real-Time PCR and Western blot analysis, respectively.

**Figure 1:**
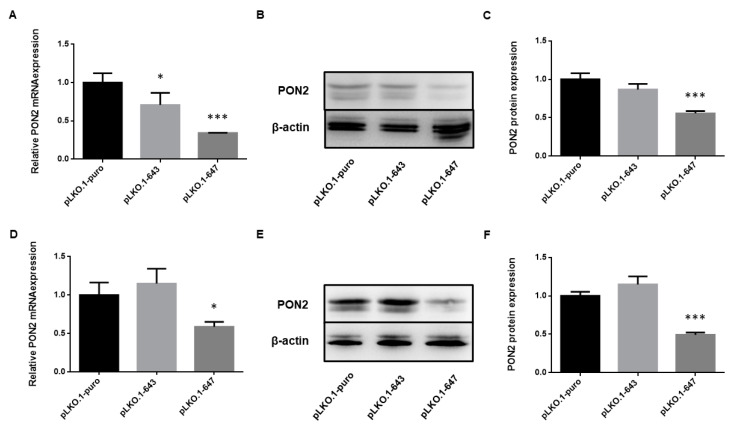
Evaluation of PON2 silencing in OSCC cell lines. HSC-3 (**A**–**C**) and HOC621 (**D**–**F**) cells were transfected with plasmids coding shRNAs targeting PON2 (pLKO.1-647 or pLKO.1-647) and with empty vector (pLKO.1-puro). Enzyme expression was then evaluated at mRNA and protein level, by Real-Time PCR (**A**,**D**) and Western blot (**B**,**E**), followed by densitometry (**C**,**F**). Values are expressed as mean ± standard deviation (* *p* < 0.05; *** *p* < 0.005).

Real-time PCR analyses revealed a significant decrease of PON2 mRNA levels in HSC-3 cells transfected with pLKO.1-643 (0.7078 ± 0.1576; *p* = 0.0475) and pLKO.1-647 (0.3435 ± 0.0013; *p* = 0.0010) compared with control (1.0000 ± 0.1220; pLKO.1-puro) (Figure 1A). In HOC621 cell line, treament with pLKO.1-647 (0.5864 ± 0.0660; *p* = 0.0352) only, but not with pLKO.1-643 (1.150 ± 0.1932; *p* = 0.4864), led to a significant reduction of enzyme expression compared with reference sample (1.0000 ± 0.1631; pLKO.1-puro) (Figure 1D).

Western blot analyses, coupled with densitometry of immunoreactive bands, demonstrated a markedly decreased PON2 protein expression for HSC-3 cells transfected with pLKO.1-647 (0.5564 ± 0.0301; *p* = 0.0004) with respect to control (1.0000 ± 0.0789; pLKO.1-puro) (Figure 1B,C). Similarly, HOC621 cells treated with pLKO.1-647 (0.4928 ± 0.0289; *p* = 0.0003) displayed a significant reduction in PON2 protein levels compared with those receiving pLKO.1-puro (1.0000 ± 0.0535) (Figure 1E,F). In both cell lines, treatment with pLKO.1-643 did not result in any relevant decrease of PON2 protein expression (0.8672 ± 0.0733 and *p* = 0.0994 for HSC-3; 1.1490 ± 0.1056 and *p* = 0.0900 for HOC621) compared with respective controls (Figure 1B,C,E,F). Considering the remarkable silencing effect induced by pLKO.1-647, cells treated with this plasmid vector, but not those transfected with pLKO.1-643, were selected for further experiments.

### 2.2. Effect of PON2 Downregulation on Viability, Proliferation and Apoptosis Induction of OSCC Cells

To explore the potential contribution of PON2 in OSCC cell metabolism, as well as to investigate the phenotypic effects associated with the induction of enzyme knockdown, cell viability and proliferation were subsequently evaluated at different time points: 0, 24, 48 and 72 h for Trypan Blue assay in both cell lines, as well as for MTT assay in HOC621 cells, while 0, 24, 48, 72 and 96 h for MTT assay in HSC-3 cells.

**Figure 2:**
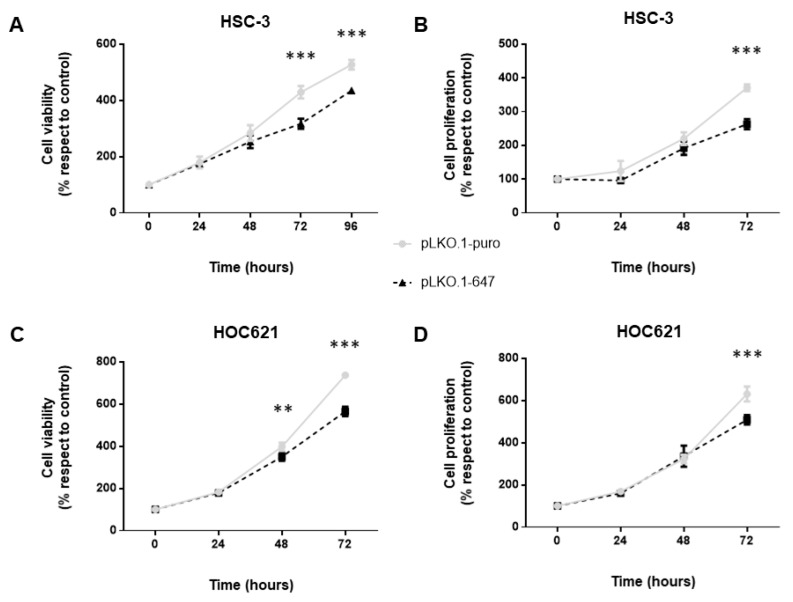
In vitro effect of PON2 knockdown on cell viability and proliferation. Cell viability was measured by performing MTT (**A**,**C**) and trypan blue exclusion (**B**,**D**) assays, in both HSC-3 and HOC621 cell lines. Values are expressed as mean ± standard deviation (** *p* < 0.01; *** *p* < 0.005).

In particular, cell viability was monitored by using the colorimetric MTT assay and results obtained clearly evidenced a significant (*p* < 0.0001) reduction of percentage values at timepoint 72h in cells downregulating PON2 (pLKO.1-647) with respect to those of control cells (pLKO.1-puro), for both cell lines (Figure 2A,C).

Concerning cell proliferation, data collected from the trypan blue exclusion assay revealed that PON2 silencing led to a significant (*p* < 0.0001) decrease of cell growth of both HSC-3 and HOC621 treated with pLKO.1-647 compared to that of reference samples (pLKO.1-puro), at 72h timepoint (Figure 2B,D).

**Figure 3:**
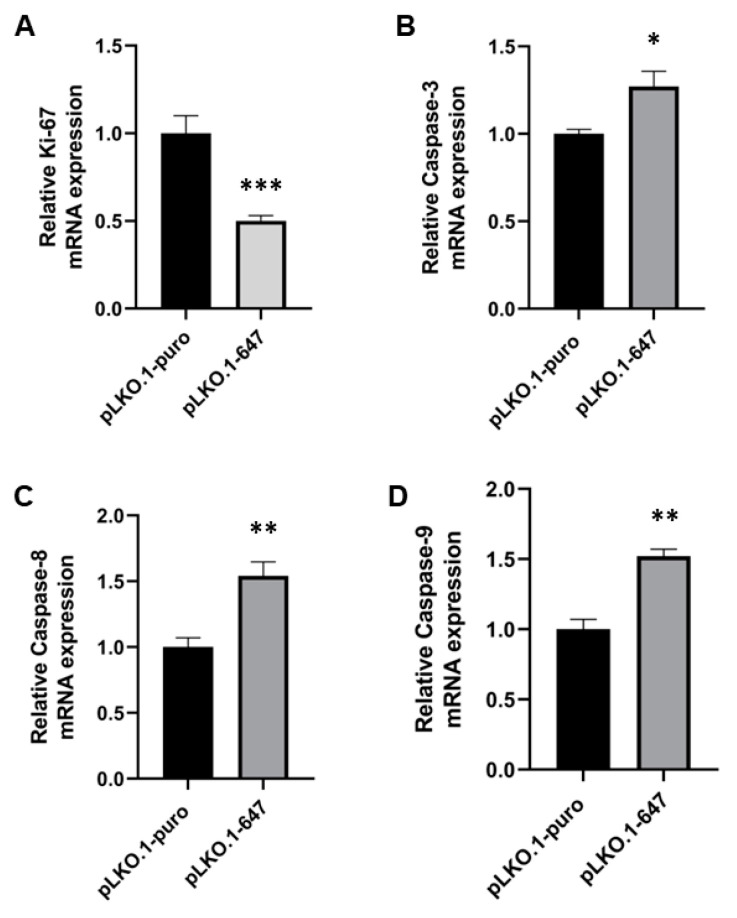
Real-Time PCR analysis of cell proliferation and apoptosis induction in HSC-3 cells, following PON2 silencing. Expression of Ki-67 (**A**), Caspase-3 (**B**), -8 (**C**) and -9 (**D**) was evaluated in control (pLKO.1-puro) and PON2 downregulating (pLKO.1-647) cells. Values are expressed as mean ± standard deviation (* *p* < 0.05; ** *p* < 0.01; *** *p* < 0.005).

Interestingly, PON2 silencing was significantly (*p* < 0.05) associated with decreased levels of Ki-67, as well as with the induction of Caspase-3, -8 and -9 expression (Figure 3).

### 2.3. Impact of PON2 Knockdown on Sensitivity of OSCC Cell Lines to Treatment with CDDP and 5-FU

The effect of PON2 silencing on response to treatment with chemotherapeutic drugs of HSC-3 and HOC621 cells was evaluated in terms of cell viability by MTT assay. Viability of cells grown in medium containing 0.1% dimethyl sulfoxide (DMSO), which was used to dissolve 5-FU, did not significantly differ from that of cells cultured with complete medium only.

**Figure 4:**
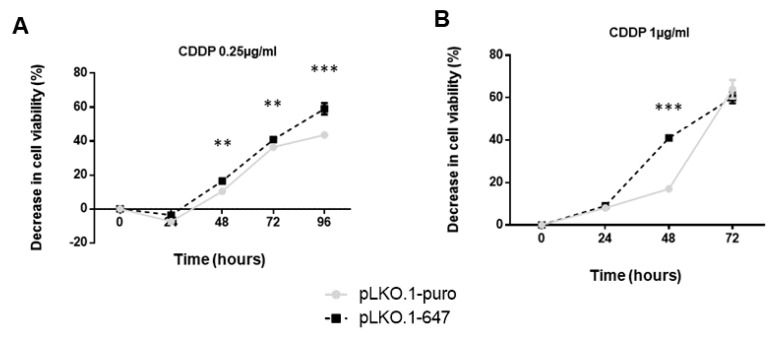
Impact of chemotherapeutic drugs on cell viability of OSCC cells. MTT assay was used to evaluate the effect of CCDP treatment on cell viability of PON2 downregulating cells (pLKO.1-647) and controls (pLKO.1-puro), related with HSC-3 (**A**) and HOC621 (**B**) cell lines. Measurements were performed at 0, 24, 48, 72 and 96 h for HSC-3 and at 0, 24, 48 and 72 h for HOC621. All values are expressed as mean ± standard deviation (** *p* < 0.01; *** *p* < 0.005).

Interestingly, treatment with 0.25 µg/mL CDDP led to a significant (*p* < 0.0001) decrease of cell viability in PON2 downregulating HSC-3 (pLKO.1-647) cells compared with that detected in control cells (pLKO.1-puro), at 96h timepoints (Figure 4A). Concerning HOC621 cell line, a significant (*p* < 0.0001) reduction of cell viability was evident 48 h after starting treatment with 1 µg/mL CDDP. However, prolonged time exposure (72 h timepoint) to treatment with this compound resulted in an excessive cell death stimulus in both pLKO.1-647 and pLKO.1-puro samples, thus preventing to appreciate potential differences in cell viability (Figure 4B).

For both cell lines, at all tested timepoints, 5-FU alone or combined with CDDP did not exert any significant difference in decrease of cell viability of pLKO.1-647 compared with pLKO.1-puro, thus demonstrating that enzyme knockdown had no significant impact on sensitivity of OSCC cells to these treatments.

**Figure 5:**
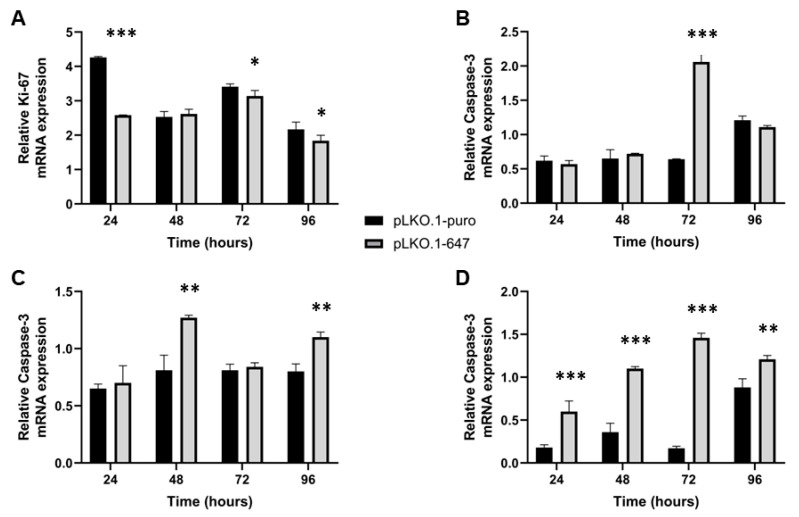
Real-Time PCR analysis of cell proliferation and apoptosis induction in HSC-3 cells, treated with CDDP. Expression of Ki-67 (**A**), Caspase-3 (**B**), -8 (**C**) and -9 (**D**) was evaluated in control (pLKO.1-puro) and PON2 downregulating (pLKO.1-647) cells. Values are expressed as mean ± standard deviation (* *p* < 0.05; ** *p* < 0.01; *** *p* < 0.005).

In HSC-3 cells treated with CDDP, PON2 knockdown led to a significant (*p* < 0.05) reduction of Ki-67 expression (at 24, 72 and 96 h) and to an increase of Caspase-3 (at 72 h), -8 (at 48 and 96 h) and -9 (at 24, 48, 72 and 96 h) levels (Figure 5).

### 2.4. FTIRM Data Analysis of Effects Induced by CDDP Treatment on PON2-Silenced and Control OSCC Cell Lines

FTIRM was exploited to evaluate more deeply the effect of the CDDP treatment on both HSC-3 and HOC621 cells after PON2 knockdown, with respect to controls. Mean IR spectra of HSC-3 after treatment with 0.25 μg/mL CDDP at 24, 48, 72 and 96 h and HOC621 after treatment with 1 μg/mL CDDP at 24, 48 and 72 h were reported in Supplementary Materials (Figures S1 and S2, respectively).

The spectral analysis was mainly focused on the evaluation of the oxidative stress caused by CDDP treatment on PON2-silenced (pLKO.1-647) and control (pLKO.1-puro) cells. In this regard, the following spectral parameters were analyzed: A_3010_/A_2960_, calculated as ratio between the areas of the bands at ~3010 cm^−1^ (vibrational modes of unsaturated CH groups in lipid alkyl chains [25]) and ~2960 cm^−1^ (vibrational modes of CH_3_ groups in lipid alkyl chains [26,27,28]), representing the unsaturation rate in lipids; A_2925_/A_2960_, calculated as ratio between the areas of the bands at ~2925 cm^−1^ (vibrational modes of CH_2_ groups in lipid alkyl chains [26,27,28]) and ~2960 cm^−1^ (vibrational modes of CH_3_ groups in lipid alkyl chains [26,27,28]), related to the presence of apoptotic processes; A_1740_/A_1655_, calculated as ratio between the areas of the bands at ~1740 cm^−1^ (vibrational modes of C=O ester groups in lipids [28,29]), and ~1655 cm^−1^ (Amide I band of proteins [28,29,30]), representing the oxidation rate, and A_1170_/A_1655,_ calculated as ratio between the areas of the bands at ~1170 cm^−1^ (non-hydrogen bonds of C-O stretching vibrations attributed to protein components such as serine, threonine and tyrosine residues [31]), and ~1655 cm^−1^ (Amide I band of proteins [28,29,30]), representing the relative amount of non-hydrogen bonded proteins.

**Figure 6:**
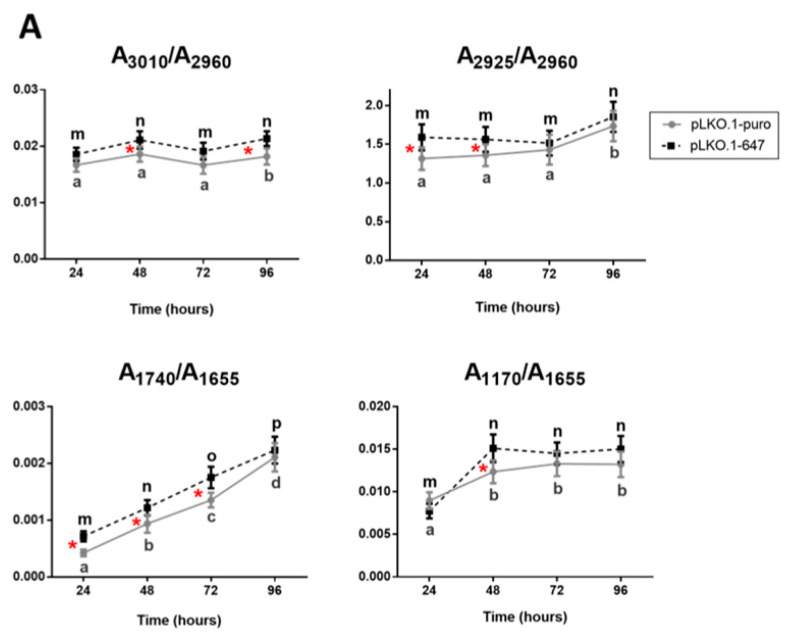

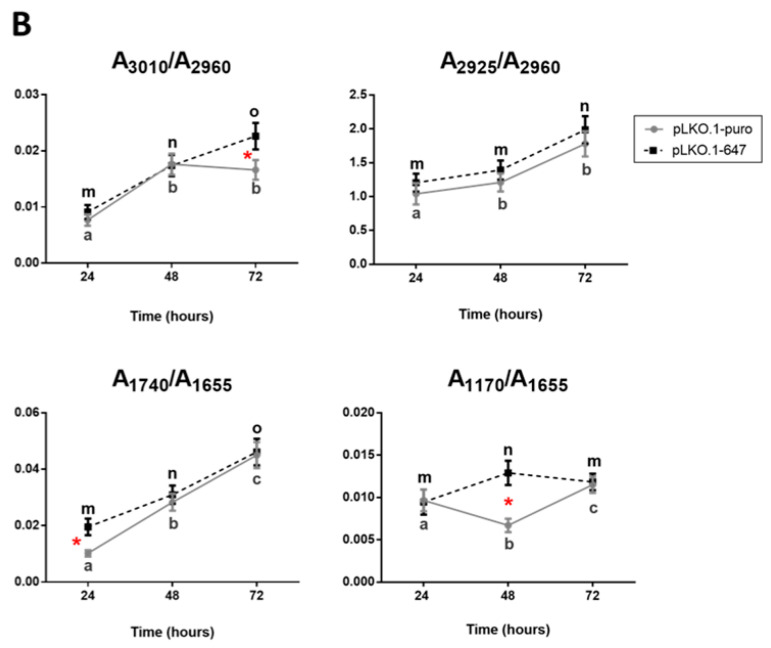
FTIRM analysis of CDDP-treated OSCC cell lines upon PON2 silencing. Statistical analysis of the following spectral markers, obtained for HSC-3 (**A**) and HOC621 (**B**) cell line: A_3010_/A_2960_ (unsaturation rate of lipids); A_2925_/A_2960_ (saturation rate of lipids); A_1740_/A_1655_ (oxidation rate of lipids), and A_1170_/A_1655_ (non-hydrogen bonded proteins). Data are presented as mean ± SD. Different letters indicate statistically significant differences within each sample (pLKO.1-puro and pLKO.1-647) at different time points (one-way ANOVA Tukey’s multiple comparisons test). Red asterisks indicate statistically significant differences between pLKO.1-puro and pLKO.1-647at each time point (Student’s *t*-test). (* *p* < 0.05).

The statistical analysis of the above defined spectral parameters led to the following considerations. As regards HSC-3 cells (Figure 6A), (i) higher values of the ratios A_3010_/A_2960_ and A_2925_/A_2960_ were observed in PON2-silenced cells (pLKO.1-647) respect to controls (pLKO.1-puro) at all time points; (ii) an increasing time-dependent trend was found at all time points in the band area ratio A_1740_/A_1655_, with higher values in cells downregulating PON2 (pLKO.1-647) respect to reference sample (pLKO.1-puro); (iii) the A_1170_/A_1655_ ratio displayed higher values at 48, 72 and 96 h after CDDP treatment in pLKO.1-647 compared with pLKO.1-puro.

Concerning HOC621 cell line (Figure 6B), the following considerations can be reported: (i) by considering the ratio A_3010_/A_2960_, an higher value was found in PON2-silenced cells (pLKO.1-647) compared with controls (pLKO.1-puro) at 72 h, even if both cell groups showed an increasing trend from 24 h to 48 h; (ii) as regards the band area ratios A_2925_/A_2960_ and A_1740_/A_1655_, they showed an increasing time-dependent trend, with higher values in pLKO.1-647 with respect to pLKO.1-puro, at all time points; (iii) concerning the ratio A_1170_/A_1655_, both cell groups showed similar values at 24 and 72 h, while a significant difference was observed at 48 h, with cells downregulating PON2 (pLKO.1-647) displaying a higher value compared with reference specimen (pLKO.1-puro).

### 2.5. PON2 Expression Level in CDDP-Resistant and Control HOC621 Cells

In order to further speculate the relation between PON2 expression and resistance to chemotherapeutic drugs, HOC621 cells were incubated with increasing concentrations of CDDP, and chemo-resistant cell clones were selected. Subsequent Western blot analysis was used to explore enzyme levels in CDDP-resistant and control HOC621 cells.

**Figure 7:**
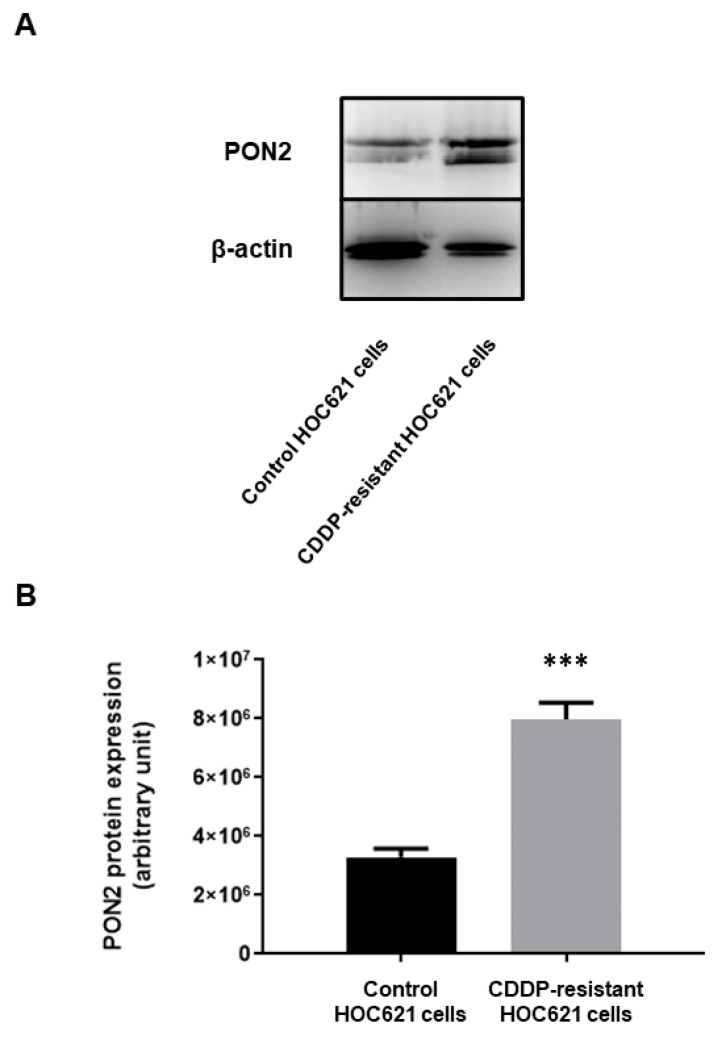
Western blot analysis of PON2 expression in control and CDDP-resistant HOC621 cells. Aliquots (20 µg) of protein extract were subjected to 12.5% SDS-PAGE and transferred to polyvinylidene fluoride membrane. Blots were probed with rabbit anti-PON2 or anti-β-actin antibodies, and analyzed with chemiluminescence (**A**). Densitometry was subsequently used to evaluate signal intensity of chemiluminescent bands and statistical analysis was performed to compare PON2 expression levels between chemo-resistant and parental cells (**B**). Values are expressed as mean ± standard deviation (*** *p* < 0.005).

Results obtained clearly demonstrated that PON2 expression was significantly (*p* = 0.001) higher (2.43-fold increase) in cisplatin-resistant compared with parental HOC621 cells (Figure 7), thus highlighting that enzyme upregulation was strictly related to CDDP resistance in oral cancer cells. This data, together with those obtained following the above reported analyses, strongly suggest that targeting PON2 could represent a promising strategy to enhance the therapeutic efficacy of CDDP during OSCC curative treatment.

**Figure 8:**
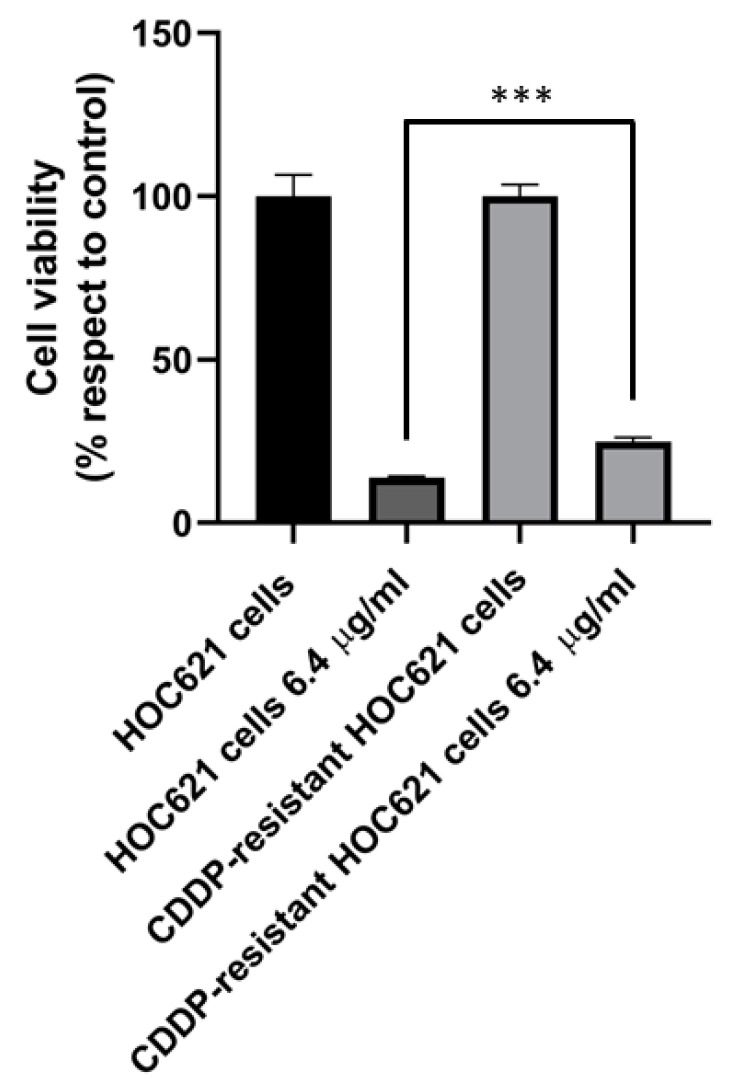
Impact of chemotherapeutic drugs on cell viability of OSCC cells. MTT assay was used to evaluate the effect of CCDP treatment (6.4 µg/mL) on cell viability of selected CDDP-resistant HOC621 and parental cells. Measurements were performed at 48 h and results were reported as percentage of the control (control equals 100% and corresponds to the absorbance value of each sample grown in DMEM at 48 h time point). All values are expressed as mean ± standard deviation (*** *p* < 0.005).

Interestingly, under treatment with 6.4µg/mL cisplatin, a significantly (*p* = 0.0007) higher decrease of cell proliferation was exhibited by parental HOC621 cells (13.82% ± 0.48%) compared to that of CDDP-resistant counterpart (24.91% ± 1.22%) (Figure 8).

## 3. Discussion

Drug resistance is mainly responsible for the efficacy failure of chemotherapeutic treatment used for management of patients affected with locally advanced or metastatic OSCC. This phenotypic trait is the result of different adaptive strategies evolved by oral cancer cell, including drug elimination or inactivation, enhanced response to DNA damage, reduction of apoptotic rate and increase of migration capacities [8].

CDDP and 5-FU, which are commonly used together with taxanes in chemotherapy regimens for oral cancer treatment, are associated with a frequency of resistance ranging between 30 and 40%, contributing to determine a poor survival of advanced stage OSCC patients [32]. Among the main mechanisms related to CDDP resistance in OSCC cells, nucleotide excision repair seems to be the main strategy acquired [33]. In particular, high expression of ERCC1 nucleotide excision repair protein is associated with CDDP resistance in HNSCC/OSCC patients [34]. Another mechanism reported in CDDP-resistant OSCC patients is the overexpression of MDR1 and MRP1, two platinum-drug efflux transporters regulated by the activation of Wnt/GSK-3β/β-catenin signaling pathway [35,36]. Regulation of apoptosis in another strategy used to counteract CDDP-mediated cytotoxicity, through overexpression of several inhibitors of apoptosis protein (IAP), such as c-IAP1, XIAP, and Survivin [37,38]. Finally, the activation of signal pathways related to Epidermal Growth Factor Receptor (EGFR), Focal Adhesion Kinase (FAK), and NF-κB exerts several activities to provide protection against CDDP-related death signals in OSCC cancer cells and are commonly reported in this subgroup of patients [33]. Regarding 5-FU, the main mechanisms related to chemoresistance are mismatch repair and base excision repair, through overexpression of uracil-DNA-glycosylase [39]. As for CDDP, another mechanism for 5-FU resistance is the inhibition of apoptosis through overexpression of IAP proteins, such as c-IAP2 and Livin [40]. Therefore, there is an urgent need to get insight into biochemical determinants and molecular mechanisms promoting the development of drug resistance, in order to improve the efficacy of chemotherapeutic treatment, as well as patient prognosis.

In the present study, the role played by PON2 in oral carcinogenesis was explored, focusing on function exerted by the enzyme in acquisition of chemoresistance by OSCC cell. PON2 silencing in oral cancer cell lines led to a significant reduction of both cell proliferation and viability, to the induction of apoptosis pathway, as well as to a marked enhancement of sensitivity to CDDP treatment. As revealed by FTIRM data, PON2 downregulating cells were less efficient in counteracting CDDP-induced oxidative damage exerted on lipids and proteins compared to controls. Interestingly, enzyme levels, as well as cell proliferation, were significantly increased in cisplatin-resistant HOC621 cells compared with controls.

Based on studies carried out in the last two decades, the antioxidative and antiatherogenic functions exerted by PON2 have been extensively elucidated and consolidated. On the contrary, only recent discoveries illustrated PON2 capacity to modulate the programmed cell death in cancer, by negatively influencing the activation of intrinsic apoptotic pathway. The execution of this pathway is finely regulated by those Bcl-2 proteins able to open the mitochondrial pores, allowing cytochrome C release into cytoplasm. However, this cascade undergoes an upstream modulation, triggered by intramitochondrial oxidative stress, that leads to cardiolipin peroxidation and consequent disruption of its binding with cytochrome C. Due to PON2 capacity to interact with coenzyme Q10 and reduce superoxide anion production, it is conceivable to attribute to the enzyme an important antiapoptotic role, thanks to its ability to decrease cardiolipin peroxidation and cytochrome C release [41]. Therefore, it is reasonable to hypothesize that PON2 upregulation featuring cancer could represent a strategy adopted by tumor cell to escape the effects induced by chemotherapeutic drugs, especially those whose mechanism of action is mediated by oxidative stress induction.

5-FU rapidly enters the cell through the same facilitated transport mechanism used by its structural analogue uracil. Inside the cell, it undergoes enzymatic conversion to active antimetabolites fluorouridine triphosphate (FUTP), fluorodeoxyuridine triphosphate (FdUTP) and fluorodeoxyuridine monophosphate (FdUMP), which negatively interfere with RNA and DNA synthesis, as well as with catalysis exerted by thymidylate synthase (TS). 5-FU cytotoxic effect is partly triggered by the misincorporation of fluoronucleotides FUTP and FdUTP into RNA and DNA, respectively, thus leading to disruption of structural integrity of these molecules. Moreover, 5-FU is able to inhibit TS enzyme activity, that is responsible for the conversion of deoxyuridine monophosphate (dUMP) to deoxythymidine monophosphate (dTMP), which in turn is further used to synthesize deoxythymidine triphosphate (dTTP). Therefore, the reduction of dTMP levels results in dTTP depletion, thus inducing imbalances in deoxynucleotide pool that is able to compromise DNA synthesis and repair processes, until causing DNA damage lethal for cell survival [42].

Regarding CDDP, once inside the cell, it is activated through chemical interaction with water, acquiring a potent electrophilic character that allows it to rapidly react with proteins and nucleic acids. DNA damage induced by treatment with CDDP is exerted thanks to its ability to bind to the N7 of purine bases, thus leading to cell cycle arrest and apoptosis induction. Besides DNA damage, CDDP cytotoxic effect can also be induced by oxidative stress, through massive ROS release that results in the activation of apoptotic pathway [43]. In the light of the above reported mechanisms of action, it is reasonable to understand why PON2 knockdown led to significant decrease of cell viability of OSCC cells treated with CDDP, whereas it seemed to display no impact of efficacy of 5-FU treatment.

As previously reported, PON2 was found to be overexpressed in several malignancies. However, the significance of enzyme upregulation, together with the impact on tumor cell phenotype associated with PON2 dysregulation, is far to be completely disclosed. A few studies have been recently carried out, aiming to speculate these aspects.

In GC cell lines, short interfering RNA (siRNA)-mediated PON2 silencing was achieved, and effect induced on cancer cell phenotype was explored. Results obtained showed that PON2 knockdown led to a decrease of cell viability, migration and invasive capacity [22].

PON2 involvement and function have been extensively analyzed in association with BC. To this aim, enzyme knockdown and overexpression were induced in T24 BC cell line and subsequent phenotypic alterations were explored. Data reported clearly demonstrated that PON2 was able to promote viability and migration of BC cell. Moreover, modulation of enzyme expression was positively correlated with chemoresistance of T24 cells to treatment with cisplatin and gemcitabine. This effect was based on the ability of PON2 to counteract oxidative stress and apoptosis, by lowering ROS production and caspase activation, respectively [44].

Enzyme overexpression in immortalized human vascular endothelial cell line EA.hy 926 treated with anthracycline doxorubicin was associated with reduction of ATP decrease and inhibition of caspase 3 activation. Moreover, apoptosis induced by treatment with staurosporine or actinomycin D was markedly decreased in EA.hy 926 cells upregulating PON2. Subsequently, enzyme effect on the efficacy of compounds used in targeted cancer therapy was also explored. Interestingly, PON2 knockdown enhanced apoptosis-related death of chronic myeloid leukemia K562 cells receiving treatment with Bcr-Abl tyrosine-kinase inhibitor imatinib, while the opposite effect was induced upon enzyme upregulation [45].

Different OSCC cell lines were found to display variable enzyme levels. When irradiated, PON2 expression was induced, being cellular response negatively correlated with endogenous level of the enzyme. In addition, radiation treatment was able to induce caspase 3/7 activation, whose extent was inversely related to PON2 basal level, with the lowest caspase 3/7 activity detected in OSCC cell lines displaying the highest endogenous enzyme expression. Further siRNA-mediated PON2 silencing was found to be associated with promotion of radiation-induced apoptosis of OSCC cells. Taken together, these results strongly suggest a potential involvement of PON2 in molecular events increasing radioresistance of OSCC cells, by protecting them from apoptotic damage caused by radiation treatment [24]. Further analyses revealed that the induction of PON2 expression in OSCC cells was found to be positively regulated by the Lymphoid Enhancer Binding Factor 1 (Lef-1), through the activation of Wnt/GSK3β/β-catenin pathway. Subsequent evaluation of enzyme levels was carried out in tumor and normal oral mucosa of a small group of OSCC patients, who were followed up for 3 years and monitored for relapse appearance. Results showed that differential PON2 expression (tumor versus normal tissue) was significantly higher in subjects with relapse disease with respect to patients who experienced relapse-free survival, thus attributing to the enzyme an interesting predictive power of disease progression [46].

The effect of shRNA-mediated PON2 silencing was recently evaluated on viability, proliferation, chemosensitivity and ROS production of A375 melanoma cells. Results showed that the induction of enzyme downregulation was significantly associated with a decrease of cell proliferation and viability, together with an increase of sensitivity of A375 cells to treatment with CDDP. Moreover, PON2 knockdown led to a significant enhancement of ROS formation in cells subjected to chemotherapeutic treatment [47].

In addition to apoptosis, other pathways or cellular processes featuring cancer cell started to be explored, in order to discover PON2 potential involvement. The study by Nagarajan et al. reported PON2 upregulation in PDAC cells, in which the enzyme was found to contribute to enhancement of glucose metabolism. Authors demonstrated that PON2 was negatively regulated by tumor suppressor p53, at transcriptional level. Since most of PDACs exhibit lack of functional p53, enzyme expression resulted markedly increased in association with this neoplasm. Under these conditions, PON2 is able to interact with glucose transporter GLUT1, facilitate PDAC cell glucose uptake and therefore increase the efficiency of glucose metabolism. These traits are partly responsible for elevated pancreatic cancer cell aggressiveness since they promote both cell growth and metastatic potential [21].

Results obtained in this study seem to suggest that PON2 could be somehow involved in mechanisms promoting oral cancer cell resistance to chemotherapy, through the regulation of fundamental processes such as cell proliferation and apoptosis. These evidences strongly support the hypothesis to target PON2 for the setup of effective molecular therapies used to treat this neoplasm. Regarding this perspective, our research group is currently focused on cloning, expression and purification of human recombinant enzyme, in order to obtain a protein preparation suitable for crystallization and further X-ray diffraction, aimed to three-dimensional structure resolution. Indeed, PON2 crystal structure is still lacking and its discovery would allow the identification of the main amino acid residues of the active site involved in catalysis and the subsequent disclosing of the enzyme kinetic mechanism, thus leading to design, synthesis and validation of potential PON2 inhibitors. In this light, further studies will clarify whether the combination of enzyme inhibitors and cisplatin could enhance sensitivity of oral cancer cells to chemotherapeutic treatment.

To the best of our knowledge, this is the first study to illustrate PON2 ability to promote cell viability, proliferation and resistance to chemotherapy in the context of this neoplasm. However, further analyses will have to be performed, in order to clarify molecular mechanisms through which the enzyme could participate to oral tumorigenesis, thus candidating PON2 as an interesting target for the effective treatment of OSCC.

## 4. Materials and Methods

### 4.1. Cell Lines and Culture Conditions

The human OSCC cell lines HSC-3 and HOC621 were kindly provided by Prof. Lorenzo Lo Muzio, Department of Clinical and Experimental Medicine, University of Foggia, Foggia, Italy. Cells were routinely cultured in Dulbecco’s Modified Eagle’s Medium/Nutrient Ham’s Mixture F-12, supplemented with 10% fetal bovine serum and 50 μg/mL gentamicin at 37 °C with 5% CO_2_.

### 4.2. PON2 shRNA-Mediated Gene Silencing

HSC-3 and HOC621 cells were seeded in 24-well plates (7 × 10^4^ cells/well) and grown in complete medium in order to achieve approximately 80% confluence at the time of transfection. The day after, cells were transfected with plasmids (0.5 μg/well) encoding shRNAs targeting different regions of PON2 transcript, such as pLKO.1-643 (5′-GCACATTTCTATGCCACAAAT-3′), targeting 574-594 PON2 mRNA nucleotide sequence, and pLKO.1-647 (5′-GCTGCTCATAGGCACTTTATA-3′), targeting 1068-1088 PON2 nucleotide sequence. Control cells were treated with empty vector (pLKO.1-puro) or with transfection reagent only (mock). Transfection procedure was performed using FuGENE HD Transfection Reagent (Promega, Madison, WI, USA), according to the manufacturer’s instructions. Forty-eight hours following transfection, the medium was replaced with a new one containing puromycin (0.5 μg/mL), in order to select, for each sample, those cellular clones harboring vectors containing DNA element conferring resistance to puromycin. To this aim, the medium was replaced every 2 days, until the complete death of cells related with mock. For all subsequent experiments, puromycin resistant cells were maintained in complete selection medium. PON2 gene silencing efficiency was evaluated at mRNA and protein level by Real-Time PCR and Western blot analysis, respectively.

### 4.3. Real-Time PCR Assay

HSC-3 and HOC621 cell pellets (1 × 10^6^ cells each) were homogenized in lysis buffer, and total RNA was isolated through the SV Total RNA Isolation System (Promega, Madison, WI, USA), according to the manufacturer’s protocol. Quantity and quality of purified RNA was assessed spectrophotometrically, by measuring the absorbance at 230, 260 and 280 nm. 2 μg of total RNA were then reverse transcribed utilizing M-MLV Reverse Transcriptase (Promega, Madison, WI, USA) and random primers, for 60 min at 37 °C. cDNA was then used as a template for the subsequent Real-Time PCR analysis. The oligonucleotide sequences of primers for PON2, Ki-67, β-actin and Caspase-3, -8 and -9 are reported in Table 1. Samples were run in duplicate for 40 cycles at 95 °C for 30 s and 58 °C for 30 s, using SsoFast EvaGreen Supermix and CFX96 Real-Time PCR Detection System (Bio-Rad Laboratories, Hercules, CA, USA). The cycle number at which the first fluorescence increase above the threshold was detected was defined as threshold cycle (Ct). Hence, fold changes in relative PON2 expression were calculated by 2^−Δ(ΔCt)^ method, where ΔCt = Ct (gene of interest) − Ct (β-actin) and Δ(ΔCt) = ΔCt (pLKO.1-643 or pLKO.1-647) − ΔCt (pLKO.1-puro). Each experiment was repeated three times.

### 4.4. Western Blot Analysis

Cell pellets (2 × 10^6^ cells), obtained from experiment performed on OSCC cell lines, were suspended in 100 μL of lysis buffer (phosphate buffered saline, containing 1% Nonidet P40, 0.1% sodium dodecyl sulfate, 2 μg/mL aprotinin and 1mM phenylmethylsulfonyl fluoride) and homogenized through a 30 gauche syringe needle. After centrifugation of homogenates at 13,000× *g* for 10 min at 4 °C, supernatants were collected.

Samples containing 20 μg protein were subjected to sodium dodecyl sulfate-polyacrylamide gel electrophoresis (SDS-PAGE), using a 12.5% polyacrylamide running gel. Proteins were transferred to polyvinylidene fluoride membranes, that were further blocked for 2 h at room temperature in tris buffered saline (TBS), containing 0.1% tween-20 and 2% bovine serum albumin (BSA). After washing, membranes were incubated overnight at 4 °C with rabbit polyclonal antibody against PON2 (Sigma-Aldrich, St. Louis, MO, USA) (1:500) or with rabbit polyclonal antibody against β-actin (Sigma-Aldrich, St. Louis, MO, USA) (1:8000) diluted in TBS, containing 0.1% tween-20 and 1% BSA. After subsequent washing, blots were incubated with horseradish peroxidase (HRP)-conjugated goat anti-rabbit IgG (Thermo Fisher Scientific, Waltham, MA, USA) (1:150,000 dilution), for 1 h at room temperature. Chemiluminescence related to PON2 bands was revealed through SuperSignal West Femto Maximum Sensitivity Substrate (Thermo Fisher Scientific, Waltham, MA, USA). ChemiDoc XRS+ System (Bio-Rad Laboratories, Hercules, CA, USA) and Image Lab Software (Bio-Rad Laboratories, Hercules, CA, USA) were then used to acquire chemiluminescent bands and to quantify related signal intensity, respectively.

### 4.5. Cell Viability and Proliferation Assays

Effect of PON2 silencing on OSCC cell viability was evaluated using a colorimetric assay with 3-(4,5-dimethylthiazol-2-yl)-2,5-diphenyl tetrazolium bromide (MTT), at different time points (0, 24, 48, 72 and 96 h) in presence or absence of chemotherapeutic drugs. Cells were seeded on 96-well plates (3 × 10^3^ cells/well) and allowed to attach overnight. The day after seeding, which represented 0 h time point, the medium was discarded and replaced with a fresh one. For each time point, 10 μL of MTT reagent (5 mg/mL in phosphate buffered saline) was dissolved in 110 μL of complete medium and added to cells (100 μL/well). After incubation for 3 h at 37 °C, the medium was replaced with 200 μL of 2-propanol, to lyse cells and dissolve the insoluble formazan crystals. The amount of formazan formed during the incubation was quantified by measuring the absorbance at 570 nm using an ELISA plate reader. Results were expressed as percentage of the control (control equals 100% and corresponds to the absorbance value of each sample at 0 h time point) and presented as mean values ± standard deviation of three independent experiments performed in triplicate.

In order to verify whether PON2 silencing influenced cell proliferation ability, HSC-3 and HOC621 cells were also subjected to trypan blue exclusion assay at different time points (0, 24, 48 and 72 h). Briefly, OSCC cells were seeded on 6-well plates (1 × 10^5^ cells/well) in serum-free medium. Twenty-four hours later, representing 0 h time point, the medium was replaced with a complete one. For each time point, cells were harvested by using 500 μL trypsin and centrifuged at 300× g for 3 min. After cell pellet resuspension in 500 μL of complete medium, trypan blue was added and cells were counted using Burker’s chamber. The number of viable cells (negative to trypan blue) was expressed as percentage of the control (control equals 100% and corresponds to the number of viable counted cells of each sample at 0 h) and presented as mean values ± standard deviation. Each experiment was performed in triplicate and independently repeated three times.

### 4.6. Chemotherapeutic Treatment

Concerning the experiments to evaluate the impact of PON2 silencing on cell sensitivity to chemotherapy, CDDP and 5-FU were selected since representing two of the most used chemotherapeutic drugs used for OSCC treatment. CDDP stock (Sigma-Aldrich, St. Louis, MO, USA) was dissolved and further diluted in culture medium, while 5-FU powder was resuspended in DMSO and added to the complete medium, keeping a constant value of final DMSO concentration (0.1%) for each treatment. OSCC cells, both those downregulating PON2 and controls, were treated with CDDP (0.1–2 µg/mL) or 5-FU (0.1–0.5 µg/mL) alone or with combinations of both compounds. Final drug concentration values, used in these series of experiments, were selected according to previous studies [48,49]. For each sample, sensitivity to drugs was evaluated through MTT assay and represented as percentage of decrease in cell viability.

### 4.7. Fourier Transform InfraRed Microspectroscopy Analysis

Based on MTT results, the influence of PON2 knockdown in both HSC-3 and HOC621 cells on response to CDDP treatment was evaluated by FTIRM. Both OSCC cell lines were seeded on 6-well plates (1 × 10^5^ cells/well) and allowed to attach overnight. The day after, the medium was discarded and replaced with a new one containing CDDP at different concentrations. In particular, HSC-3 cells were treated with 0.25 μg/mL drug for 24, 48, 72 and 96 h, while HOC621 received treatment with 1 μg/mL drug for 24, 48 and 72 h. At each time point, cells were harvested by trypsinization, centrifuged at 500× *g* for 3 min at +4 °C, washed with isotonic saline solution (0.9% NaCl) and centrifuged again. Cell pellets underwent fixation by resuspension and incubation with 500 μL 4% paraformaldehyde for 1h. After centrifugation, samples were washed three times with 500 μL 0.9% NaCl and resuspended in this solution (500 μL) until FTIRM measurements. Each experiment was performed in triplicate and independently repeated three times.

A Bruker INVENIO interferometer, coupled with a Hyperion 3000 Vis-IR microscope and equipped with a HgCdTe (MCT_A) detector operating at liquid nitrogen temperature (Bruker Optics, Ettlingen, Germany), was used. Just before acquisitions, cell samples were centrifuged, resuspended in 500 μL MilliQ water and centrifuged twice at 800× *g* for 10 min. An aliquot of 15 μL was collected from each sample, dropped onto a CaF_2_ optical window (13 mm-diameter and 1mm-thick) and left to air-dry. On each drop, ~60 microareas (30 × 30 µm), containing groups of 3–4 densely packed cells, were selected by the television camera, and the corresponding IR spectra were collected in transmission mode in the 4000–800 cm^−1^ MIR region (512 scans, spectral resolution 4 cm^−1^, zero-filling factor 2, scanner velocity 40 kHz). Before each measurement, the spectrum of the background was acquired on a clean portion of the CaF_2_ window by using the same parameters [50,51].

Raw spectra were processed as follows. All IR spectra showing a peak height at ca. 1655 cm^−1^ lower than 0.06 a.u. were discarded. Then the remaining spectra were submitted to Atmospheric Compensation and Vector Normalization routines on the full spectral range (OPUS 7.5, Ettlingen, Germany) to avoid contributions from atmospheric carbon dioxide and water vapor and to correct tiny differences in samples’ thickness. On these pre-processed spectra, the following analyses were performed. For each experimental group, the mean absorbance spectrum and the corresponding mean absorbance spectra ± standard deviation (SD) spectra were calculated, baseline linear fitted and then curve fitted in the 3050–2800 cm^−1^ (representative of cellular lipids) and 1800–900 cm^−1^ (representative of proteins, carbohydrates, and nucleic acids) spectral regions. The underlying bands were selected based on second derivative analysis and fixed before running the iterative process to obtain the best reconstructed curve (residual close to zero; bandwidth 10 to 40 cm^−1^ range) (GRAMS/AI 9.1, Galactic Industries, Inc., Salem, NH, USA). For each underlying band, the position (expressed in wavenumbers, cm^−1^) and the integrated area were defined and used to calculate specific spectral parameters reported in the Section 2, according to literature [25,26,52].

### 4.8. Development of CDDP-Resistant OSCC Cell Clones

A pulsed-selection strategy was adopted, where cells were treated with chemotherapeutic agent and then allowed to recover in a drug-free medium [48,53]. To this purpose, HOC621 cells were seeded onto a 24-well plate at 20% confluence. Following overnight culture, cells were incubated with 0.1 μg/mL cisplatin for 48 h. Upon medium replacement with a new one containing no chemotherapeutic drug, cells were allowed to grow until reaching 100% confluence. Subsequently, cells were subcultured onto another 24-well plate at 20% confluence and subjected to incubation with a higher cisplatin concentration (0.2 μg/mL), followed by recovery in a CDDP-free medium. This protocol was repeated by using increasing concentrations of cisplatin (0.4, 0.6, 0.8 and 1 μg/mL) each time. Cells that were able to survive and grow in a medium containing 1 μg/mL cisplatin were selected and designated as CDDP-resistant HOC621 cells. Further evaluation of PON2 protein expression in chemo-resistant and control cells was carried out through Western blot analysis.

In order to speculate whether CDDP-resistant HOC621 cells displayed different proliferation rating compared with controls, both samples were subjected to MTT assay, in absence or in presence of cisplatin at different concentrations, ranging between 0.05 and 6.4 μg/mL.

### 4.9. Statistical Analysis

GraphPad Prism software version 8.00 for Windows (GraphPad Software, San Diego, CA, USA) was used to perform statistical analyses on obtained data. Student’s t-test, one-way and two-way analysis of variance (ANOVA), Tukey’s multiple comparisons test and the Mann–Whitney U test were adopted to evaluate differences among examined samples. Statistical significance was set at *p* < 0.05.

## Figures and Tables

**Table 1:** Primers used for quantitative Real-Time PCR.

Target Gene	Sequence
PON2	Forward 5′-TCGTGTATGACCCGAACAATCC-3′ Reverse 5′-AACTGTAGTCACTGTAGGCTTCTC-3′
Ki-67	Forward 5′-GACATCCGTATCCAGCTTCC-3′ Reverse 5′-CCGTACAGGCTCATCAATAAC-3′
Caspase-3	Forward 5′-TGGAACCAAAGATCATACATGG-3′ Reverse 5′-CAGACCGAGATGTCATTCCA-3′
Caspase-8	Forward 5′-GATGATGACATGAACCTGCTG-3′ Reverse 5′-TTTGCTGAATTCTTCATAGTCGTT-3′
Caspase-9	Forward 5′-TACTTTCCCAGGTTTTGTTTCC-3′ Reverse 5′-AAAGCAACCAGGCATCTGTT-3′
β-actin	Forward 5′-TCCTTCCCTGGGCATGGAGT-3′ Reverse 5′-AGCACTGTGTTGGCGTACAG-3′

## Data Availability

The data presented in this study are available on request from the corresponding author.

## Supplementary Materials

The following supporting information can be downloaded at: https://www.mdpi.com/article/10.3390/ijms24010338/s1.
