# Supplementary material for: Role Played by Paraoxonase-2 Enzyme in Cell Viability, Proliferation and Sensitivity to Chemotherapy of Oral Squamous Cell Carcinoma Cell Lines
Source: Int J Mol Sci. 2022 Dec 25;24(1):338. doi: 10.3390/ijms24010338 (PMC9820498; doi:10.3390/ijms24010338)
Supplement: Supplementary file 1 [file ijms-24-00338-s001.zip › ijms-2012495-supplementary.pdf]

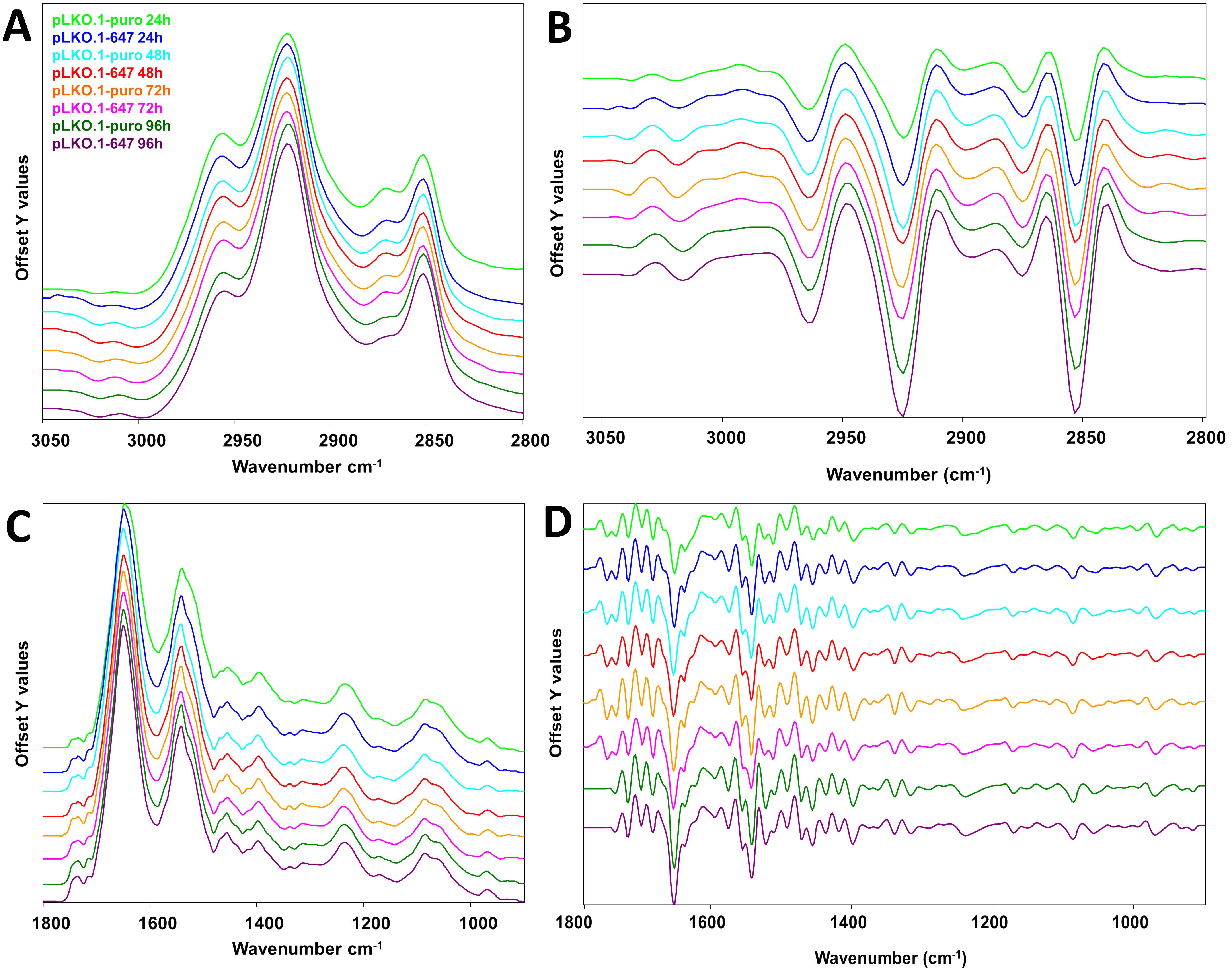

Figure S1. HSC3 IR spectra shifted along Y axis for a better comprehension and reported in 3050-2800 cm^{-1} spectral range in absorbance values **A)**, and in second derivative mode **B);** in 1800-900 cm^{-1} spectral range in absorbance values **C)**, and in second derivative mode **D)**.

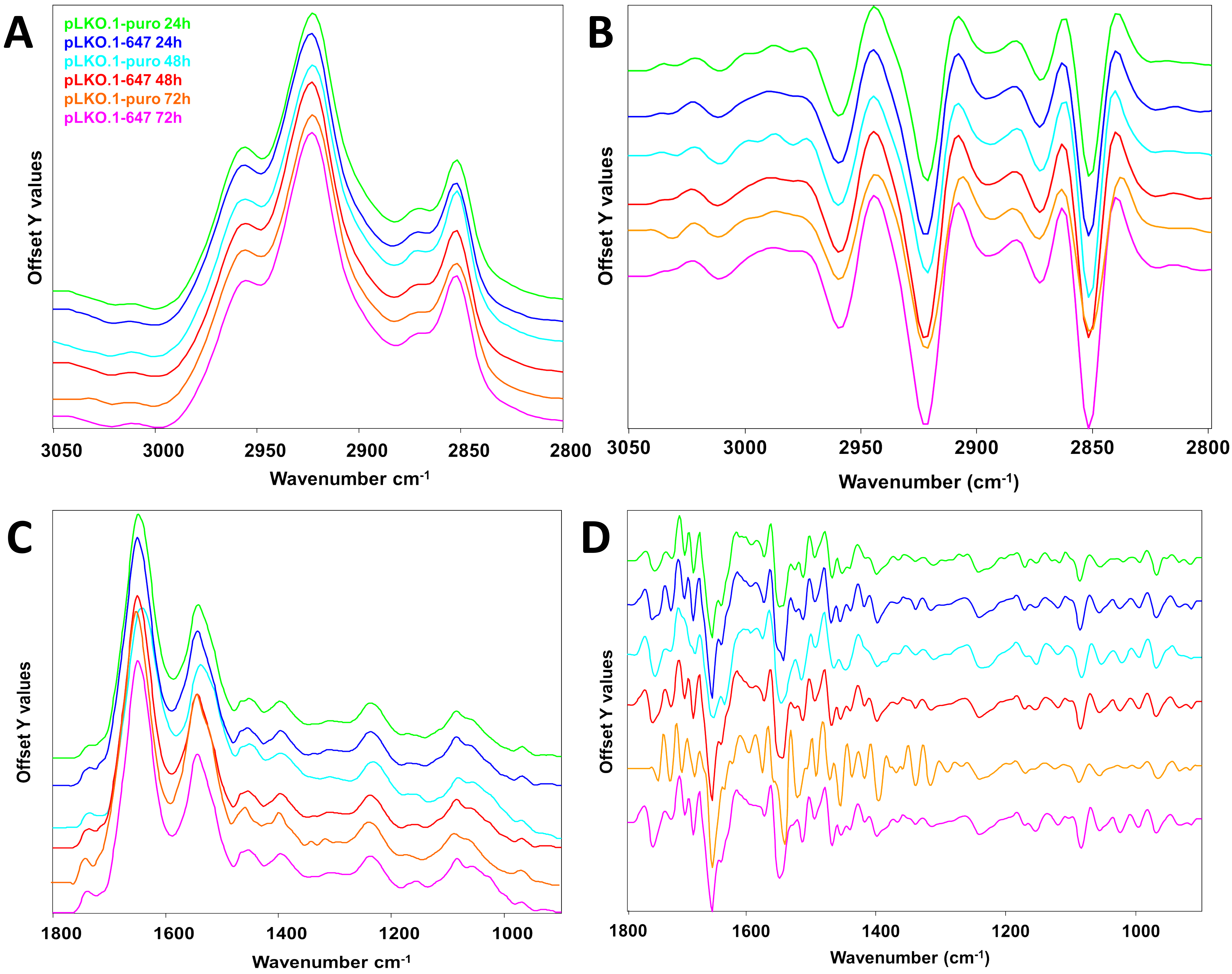

Figure S2: HOC621 IR spectra shifted along Y axis for a better comprehension and reported in 3050-2800 cm^{-1} spectral range in absorbance values **A)**, and in second derivative mode **B);** in 1800-900 cm^{-1} spectral range in absorbance values **C)**, and in second derivative mode **D)**.
